# Thyroid strain in pregnancy: a nationwide study of iodine nutrition and thyroglobulin among Faroese pregnant women

**DOI:** 10.1093/ejendo/lvaf132

**Published:** 2025-06-20

**Authors:** Herborg Líggjasardóttir Johannesen, Anna Sofía Veyhe, Pál Weihe, Maria Skaalum Petersen, Stine Linding Andersen, Stig Andersen

**Affiliations:** Department of Endocrinology and Medicine, The National Hospital of the Faroe Islands, Torshavn FO 100, Faroe Islands; Centre of Health Science, Faculty of Health Science, The University of the Faroe Islands, Torshavn FO 100, Faroe Islands; Steno Diabetes Centre Faroe Islands, The National Hospital of the Faroe Islands, Torshavn FO 100, Faroe Islands; Department of Clinical Medicine, Aalborg University, Aalborg DK 9000, Denmark; Department of Research, The National Hospital of the Faroe Islands, Torshavn FO 100, Faroe Islands; Centre of Health Science, Faculty of Health Science, The University of the Faroe Islands, Torshavn FO 100, Faroe Islands; Department of Research, The National Hospital of the Faroe Islands, Torshavn FO 100, Faroe Islands; Centre of Health Science, Faculty of Health Science, The University of the Faroe Islands, Torshavn FO 100, Faroe Islands; Department of Research, The National Hospital of the Faroe Islands, Torshavn FO 100, Faroe Islands; Centre of Health Science, Faculty of Health Science, The University of the Faroe Islands, Torshavn FO 100, Faroe Islands; Department of Research, The National Hospital of the Faroe Islands, Torshavn FO 100, Faroe Islands; Department of Clinical Medicine, Aalborg University, Aalborg DK 9000, Denmark; Department of Clinical Biochemistry, Aalborg University Hospital, Aalborg DK 9000, Denmark; Department of Clinical Medicine, Aalborg University, Aalborg DK 9000, Denmark; Arctic Health Research Centre, Aalborg University Hospital, Aalborg DK 9000, Denmark; Greenland Centre for Health Research, University of Greenland, Nuuk GL 3900, Greenland

**Keywords:** iodine, thyroglobulin, pregnancy, awareness, urinary iodine excretion, health survey, Faroe Islands, artic society

## Abstract

**Objective:**

Abnormal thyroid function is particularly problematic in pregnant women. Iodine is important to maintain normal thyroid function, and the World Health Organization (WHO) recommends a raised iodine intake in pregnant compared with non-pregnant adults. The raised iodine intake level from 100 to 150 µg/L includes a safety margin, and we hypothesized that the thyroid is not strained until urinary iodine concentration (UIC) is below 100 μg/L.

**Design:**

Nationwide cross-sectional study.

**Setting:**

Routine prenatal care at the National Hospital System of the Faroe Islands, 2020-2022.

**Participants:**

A total of 623 pregnant women, representing 63% of all pregnancies in the Faroe Islands during the study period, with no known thyroid disease.

**Exposure:**

Iodine-containining dietary intake was assessed indirectly through urinary iodine concentration (UIC) measured on spot urine using the Sandell–Kolthoff reaction.

**Main Outcome:**

The primary outcomes were serum Thyroglobulin (s-Tg) and thyrotropin (TSH) concentration serving as indicators of potential thyroid strain and thyroid function.

**Measures:**

UIC and TSH was measured in all participants. sS-Tg was measured in a randomly selected subset of 236 participants.

**Results:**

Women were seen in median gestational week 20. None had elevated TSH; the median UIC was 108 µg/L, and the median s-Tg was 10.3 µg/L. Serum Tg differed only for the group with UIC below 50 µg/L (*P* = .02), but not when UIC was above 50 µg/L. TSH increased with higher UIC (*P* < .001).

**Conclusions and Relevance:**

Thyroglobulin levels increased only in the group of Faroese pregnant women with UIC below 50 µg/L, indicating that strain on the thyroid gland was seen with low UIC levels parallel to that of non-pregnant adults. Our results suggest that the UIC limit recommended in pregnancy may be overly strict and warrant reconsideration to balance health efficacy.

## Graphical Abstract

**Graphical Abstract:**
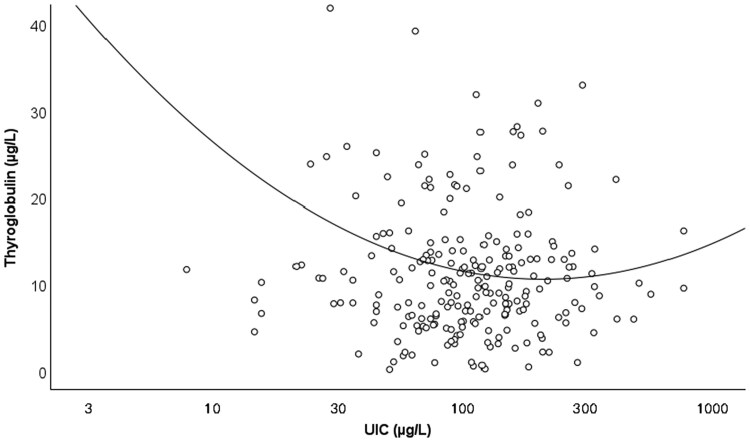

Significance Current iodine recommendations assume increased maternal thyroid vulnerability during pregnancy. This nationwide study from the Faroe Islands challenges these recommendations, as urinary iodine concentrations (UICs) had to fall below 50 µg/L before thyroid strain was observed, indicated by elevated thyroglobulin levels. These findings suggest that the currently recommended pregnancy-specific UIC threshold of 150 µg/L may be unnecessarily high. Thyroid adaptation in pregnancy may tolerate lower iodine levels than presently assumed. This has important implications for the classification of iodine deficiency and for public health policies, and it supports reconsideration of current guidelines for iodine intake in pregnancy.

## Introduction

Pregnant women of the Faroe Islands are at risk of mild iodine deficiency (ID) due to dietary changes,^1^ and such a population provides an opportunity to explore the association between thyroid function and iodine intake among pregnant women with borderline ID.

Thyroid function is critical for foetal and maternal health during pregnancy^2-4^ with iodine being an obligatory, integral component of thyroid hormone synthesis to uphold foetal brain development and growth.^5^ Despite the recognized importance of iodine for health, variations in iodine status across populations have raised concerns about potential implications for maternal and foetal health.^6^ According to the World Health Organization (WHO), iodine intake during pregnancy is considered insufficient when the median urinary iodine concentration (UIC) is below 150 µg/L, and adequate when it lies within the range of 150-250 µg/L.^7^ This WHO classification is intended for the population-level assessment and does not reflect individual iodine sufficiency. Around 90% of the ingested iodine is excreted in the urine,^8^ and the latest update on the Nordic Nutrition Recommendation has added a focus on UIC and raised the recommended iodine nutrition to align with the WHO recommendations.^9,10^

Thyroglobulin (Tg) is a thyroid-specific glycoprotein mainly found in thyroid follicles where it plays an important role in the production of thyroid hormones, their storage, and release.^11^ Under normal physiological conditions, only small amounts of Tg enter the circulation. However, increased thyroidal activity or enhanced follicular cell turnover, as seen in iodine-deficient states, may result in leakage of Tg into the bloodstream. Hence, serum Tg has been shown to reflect long-term iodine status, particularly in populations with insufficient iodine intake. Several studies have demonstrated that serum-Tg (s-Tg) concentrations increase as UIC falls below 100 μg/L, a threshold commonly used to define insufficient iodine intake in non-pregnant adults.^12,13^ While this threshold may not directly translate to pregnancy, it serves as a pragmatic reference for assessing whether similar thyroidal responses to iodine insufficiency are evident in pregnant populations, where comparable associations have been reported.^14^ This association highlights the need for comprehensive, population-based studies to explore how iodine availability may influence thyroid strain in pregnant women.

This nationwide observational study aims to clarify the link between iodine status and thyroid function in pregnant women. The primary objective was to assess whether UIC below 150 μg/L impacts thyroid function in pregnant women and to examine whether the median UIC level of 110 μg/L^1^ observed in our study population was sufficient to maintain normal thyroid function during pregnancy. Specifically, we explored how s-Tg levels differ with median UIC among pregnant women in the Faroe Islands. We speculated that the level of UIC set by the WHO may be too strict, and we hypothesized that s-Tg levels increase only when UIC is below 100 μg/L in pregnant women.

## Materials and methods

### Study design and participants

This was a nationwide cross-sectional study recruiting pregnant women from June 2020 to April 2022 when attending the routine obstetric ultrasound examinations, typically performed at median gestational week 20 (IQR: 19-21) as described in detail previously.^15^ All pregnant women attending routine obstetric check-ups during the study period were considered eligible for inclusion.

**Figure 1.**
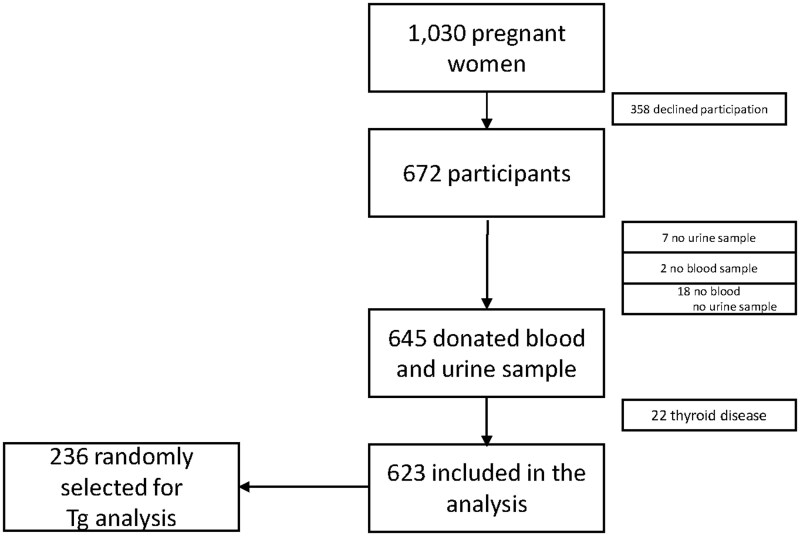
Flow chart of participation, inclusion, and exclusion.

A total of 654 individuals (63%) were willing to participate in the study. Among them, 645 (62%) donated blood and urine samples. Among these, 22 (2%) individuals were excluded from the analysis due to the use of thyroid medication, leaving 623 women (60%) with complete information. A random selection of 236 participants were chosen for the measurement of s-Tg (Figure 1). We targeted a minimum sample size of 206 serum samples to achieve a precision of 10% for s-Tg with a 95% CI.^16^

### Area of investigation

The Faroe Islands are an archipelago situated in the North Atlantic Ocean, ∼655 km to the north of the shores of Europe. The predominant economic activity in this region revolves around fishing and salmon farming. The Faroese healthcare system follows a Scandinavian model, providing tax-funded medical services available to all inhabitants. This structure ensures access to healthcare services without financial barriers for the Faroese population.

#### Questionnaires

At the time of inclusion during pregnancy, participants completed two questionnaires collecting retrospective information on lifestyle habits during the 3 months prior to conception. The first questionnaire covered age, marital status, reproductive history, education, geographical location, smoking, alcohol consumption, medication use, and body mass index (BMI).^1^ Information on preconception and present supplement use, including iodine content, was obtained through second self-administered questionnaires completed at the time of urine sampling. Information on thyroid disease was self-reported.

### Biochemical analysis of samples

The first-morning void (FMV) urine samples were collected in iodine-free polyethylene containers and stored at −80 °C until analysis. These samples were included in the routine obstetric check-ups with a first-morning urine sample for dipstick testing, thus ensuring standardized, practical, and optimal number of samples collected. All urine samples were sent for analysis at the iodine laboratory at Aalborg University Hospital, which is included in the ensuring the quality of urinary iodine procedures (EQUIP) Programme (E-117) certified by the US Centres for Disease Control and Prevention. Iodine was measured using the Sandell–Kolthoff reaction modified according to Wilson and Van Zyl.^17-19^ The principle was evaporation and alkaline ashing of the sample, followed by resuspension and measurement of iodine by spectrophotometry to detect the catalytic role of iodine in the reduction of ceric ammonium sulphate in the presence of arsenious acid.^18^ The laboratory has performed well in quality assurances over the past decades under the EQUIP Programme (E-117).^1^

The laboratory of the National Hospital in the Faroe Islands analysed thyroid function tests using a chemiluminescent microparticle immunoassay system (Alinity I; Abbott Laboratories, Longford, Ireland), thyrotropin (TSH), and free tetraiodothyronine (fT4). For TSH, the detection limit was 0.01 mIU/L. Local data on reference intervals are not available for Faroese pregnant women, and the reference range supplied by the manufacturer in non-pregnant adults was 0.3-4.9 mIU/L. In pregnant women, a single TSH measurement should be above 6.0 mIU/L to detect true abnormal thyroid function.^20^ The detection limit for free T4 (fT4) was 3.6 pmol/L, and the reference range in non-pregnant adults was 9.1-23.8 pmol/L according to the manufacturer. The analysis of s-Tg was performed at Aalborg University Hospital (Aalborg, Denmark). S-Tg was analysed using immunofluorescence analysis on Kryptor Compact (Thermo Fisher Diagnostics). The detection level was 0.2 µg/L, with a measurement range of up to 200 000 µg/L. All serum analyses were performed in the certified hospital laboratories undergoing scrutinizing quality control programmes.

### Ethical approval and data management

The Faroese Scientific Ethical Committee (February, 12, 2020) and the Faroese Data Protection Agency (reference number 20/00032-6) approved the study. Participation was voluntary, and all participants signed written informed consent forms complying with the Declaration of Helsinki.

### Statistical analysis

The distribution of continuous variables was assessed visually using histograms and Q–Q plots and numerically by evaluating skewness and kurtosis. Summary statistics are presented as means and SDs for normally distributed variables and as medians with IQRs for skewed variables. Categorical variables were presented as counts and percentages in each category.

Comparison of two groups was performed using the Student's *t*-test or Mann–Whitney test as appropriate. The ANOVA or the Kruskal–Wallis test was employed for comparisons involving multiple groups, and *χ*^2^ test was applied for categorical variables. We examined associations with correlations and standard regression models. Although a few s-Tg values exceeded 1.5× IQR, only one clear outlier was visually distinct and excluded based on boxplot assessment.

We explored the UIC level at which the thyroid gland was burdened, as suggested by the elevation of s-Tg. Thus, the association between s-Tg and UIC was investigated using a linear regression model. To explore potential non-linear associations, we employed restricted cubic spline regression with three knots, placed a priori at the 10th, 50th, and 90th percentiles of the marginal distribution of the exposure. Recommendations were found in regression modelling strategies.^21^ The results were obtained by squaring the predicted and the 95% CIs around the predicted mean values of s-Tg. The square root transform was chosen as this approximated the normal distribution better than the log transform did when examining the residuals.

A linear regression model was used to examine the association between TSH and UIC groups. Spearman's rank correlation coefficient (rho) was calculated to assess the correlations between UIC groups and TSH, fT4, and s-Tg.

Statistical analyses were conducted using the Statistical Package for the Social Sciences (SPSS version 28.0; SPSS Inc., Chicago, IL, USA) and R (version 2024; R Core Team.^22^ Splines were fitted using the *splines* package. Plots and figures were generated using *ggplot2* packages in R.^23^

## Results

Among the 623 women who donated urine and blood samples, 236 women were randomly selected for measurement of Tg in serum (Table 1). Participants had a median age of 30 years, and 99.6% were singleton pregnancies. According to self-report, 78% of participants were using iodine-containing supplements at the time of sampling. None of the participants had an elevated TSH (Table 1).

**Table 1.** Maternal characteristics and distribution of TSH and s-Tg.

		TSH (mIU/L)				s-Tg (µg/L)^e^			
Variable	Group	*n*	Median	IQR	*P*-value	*n*	Median	IQR	*P*-value
Age group	<25	76	1.24	0.93-1.57	0.7^c^	31	11.8	7.8-13.9	.2^c^
	25-29	192	1.24	0.83-1.56		71	10.5	6.4-15.6	
	30-34	207	1.23	0.89-1.64		74	9.6	6.0-12.5	
	35-39	107	1.20	0.82-1.66		42	7.9	5.0-13.3	
	≥40	20	1.07	0.56-1.87		7	15.6	6.9-18.4	
Age group	<35	481	1.24	0.88-1.60	**<0**.**001**^d^	179	10.5	6.6-14.1	.4
	≥35	127	1.19	0.78-1.67		49	8.0	5.3-15.4	
Parity	Nulliparous	190	1.22	0.89-1.67	0.6	69	10.7	7.0-15.0	.4
	Multiparous	423	1.23	0.82-1.58		158	9.8	6.0-13.9	
Place at birth	Faroe Islands	535	1.21	0.82-1.58	**0**.**02**	207	10.1	6.4-14.1	.9
	Outside the Faroe Islands	90	1.34	0.98-1.83		25	11.4	5.4-20.2	
Diabetes in pregnancy^a^	No	534	1.24	0.85-1.62	0.7	198	10.4	6.3-14.9	.1
	Yes	6	1.16	0.82-1.54		2	—	—	
Pre-pregnancy obesity^b^	No	437	1.23	0.89-1.63	0.3	164	10.4	6.6-13.7	.9
	Yes	142	1.17	0.78-1.60		57	11.26	5.6-15.8	
Smoking or snuff in pregnancy	No	549	1.23	0.89-1.62	0.1	201	10.14	6.2-14.0	.4
	Yes	56	1.15	0.63-1.60		25	11.7	7.3-19.5	

Bold values indicate statistically significant differences (*P* < 0.05). Abbreviations: BMI, body mass index; s-Tg, thyroglobulin; TSH, thyrotropin.

^a^Has the general practitioner ever told you that you have diabetes (Type 1 diabetes mellitus or type 2 diabetes mellitus; not stated).

^b^BMI ≥ 30.0.

^c^Kruskal–Wallis H test.

^d^Mann–Whitney U test.

^e^One outlier was removed.

The median UIC was 108 µg/L in the entire study population and did not differ significantly from that of the subgroup with s-Tg measurement (*P* = .3). Both groups showed a parallel time trend with decreasing UIC observed over the study period. The overall median s-Tg was 10.3 µg/L.

**Figure 2.**
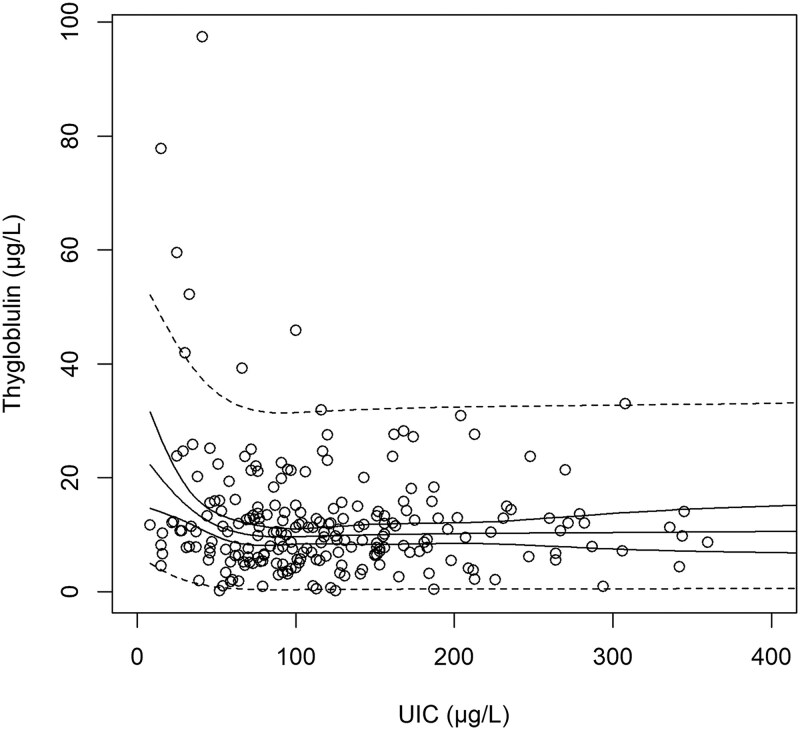
Scatterplot of thyroglobulin (s-Tg) (*y*-axis) vs urinary iodine concentration (UIC, *x*-axis). Solid lines represent the predictions and CIs of mean s-Tg as predicted by UIC. Dotted lines represent CIs of the total sample predictions.

**Figure 3.**
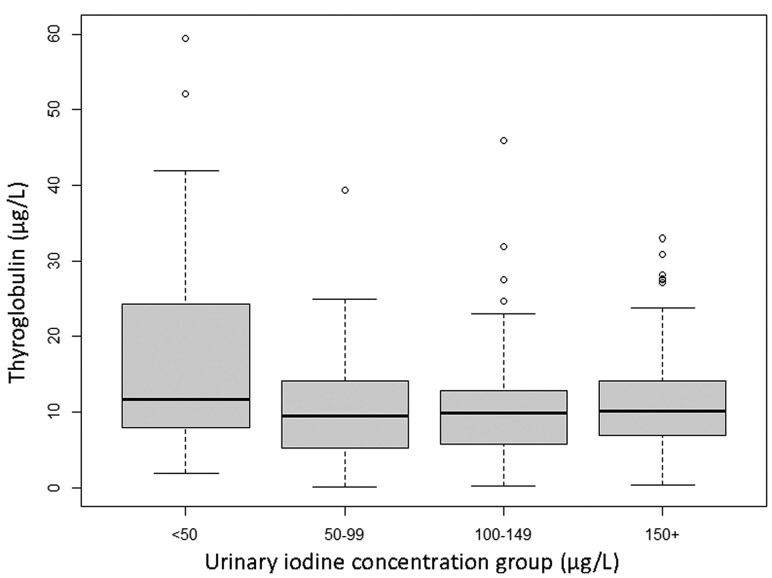
Boxplot of thyroglobulin (s-Tg) among pregnant women split into groups by urinary iodine concentration (UIC). Thyroglobulin increased when UIC was below 50 µg/L (*P* = .02). The mean values were calculated from the square root of s-Tg and then back-transformed by squaring the numbers with 95% CI. The findings were as follows: UIC <50 µg/L: 16.7 µg/L (11.4-23.0); UIC 50-99 µg/L: 9.5 µg/L (7.9-11.2); UIC 100-149 µg/L: 9.5 (7.6-11.7); and UIC ≥150 µg/L: 10.5 (9.1-12.1).

Inference was established between UIC and s-Tg using the regression model (*P* = .02, $R_{\mathrm{adj}}^{2}$ = 0.033). The lowest predicted value of s-Tg was found with UIC around 90 µg/L, and the predicted s-Tg mean value was significantly higher when UIC fell below 50 µg/L (Table 2; Figure 2). The observed s-Tg increased significantly when UIC fell below 50 µg/L (median s-Tg 9.8 vs 11.6 µg/L; *P*_MW_ = 0.02; Figure 3).

**Table 2.** Relationship between UIC and mean s-Tg levels in pregnant women as inferred from linear regression model with natural cubic splines on UIC as an independent variable, three knots at the 10th, 50th, and 90th quantiles, and the square root of s-Tg as the outcome variable.

UIC value	Predicted mean of s-TG	95% CI of mean s-Tg values	95% prediction interval of s-Tg observations
20	18.2	(13.5-23.8)^a^	(3.1-45.8)
30	15.4	(12.3-18.8)^a^	(2.0-41.3)
40	13.2	(11.0-15.6)^a^	(1.3-37.6)
50	11.7	(9.7-13.8)^a^	(0.8-35.0)
60	10.6	(8.8-12.7)	(0.6-33.2)
70	10.0	(8.3-12.0)	(0.4-32.1)
80	9.7	(8.2-11.5)	(0.4-31.6)
90	9.6	(8.2-11.1)	(0.4-31.4)
100	9.7	(8.4-11.1)	(0.4-31.4)
300	10.4	(7.5-13.7)	(0.5-32.8)

The lowest predicted s-Tg was found when UIC was 90 µg/L.

^a^s-Tg was significantly higher when UIC went below 50 µg/L compared with higher UIC values (*P* < 0.05). The 95% prediction interval is the range in which 95% of future observations are expected to fall, based on the model, and the bounds correspond to the 2.5th and 97.5th percentiles of this distribution.

Abbreviations: s-Tg, thyroglobulin; UIC, urinary iodine concentration.

We did not detect an association between s-Tg and maternal age (*P* = .3) or gestational week (*P* = .4).

**Figure 4.**
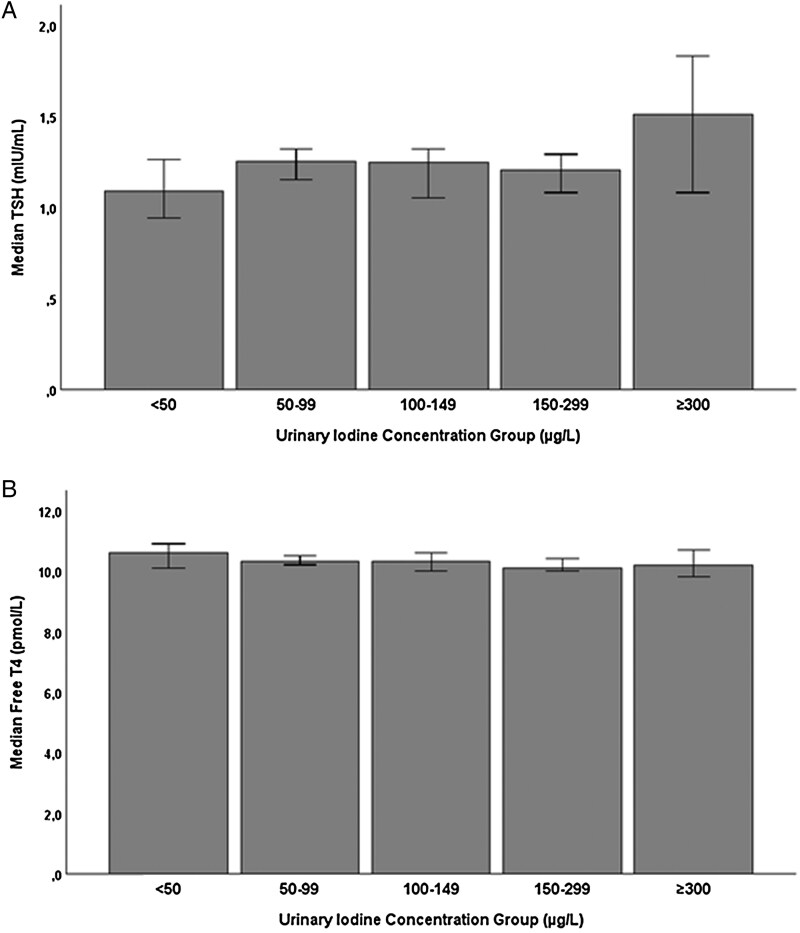
(A) Thyrotropin (TSH) among pregnant women split into groups by urinary iodine concentration (UIC). Thyrotropin showed an increasing trend with rising UIC (*P* < .001). (B) Free tetraiodothyronine (T4) split into groups by UIC. Error bars represent 95% CI.

TSH increased with higher UIC (*P* < .001) (Figure 4A) and was distinctly higher in the group with UIC above 300 µg/L compared with the group with UIC 150-299 µg/L (*P* = .007). In addition, fT4 did not differ between UIC groups (*P* = .3) (Figure 4B). Similar results were found in crude correlation analysis using continuous variables (TSH-UIC, *P* < .001; fT4-UIC, *P* = .97).

**Figure 5.**
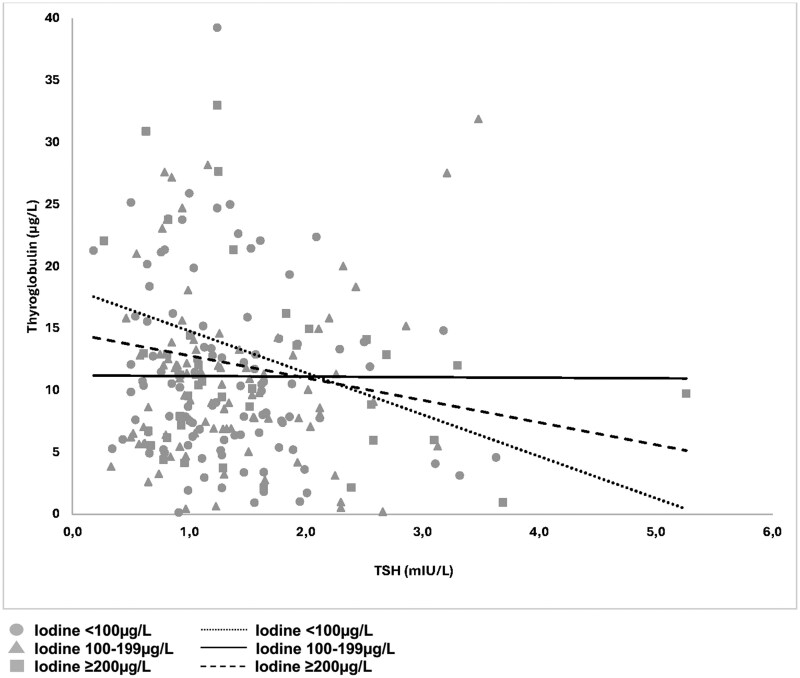
Thyroglobulin (s-Tg) among pregnant women by thyrotropin (TSH), split into three urinary iodine concentration (UIC) groups of <100, 100-199, or 200+ µg/L. Three trendlines are included for each of these groups. The solid trendline shows that s-Tg did not differ with TSH when UIC was 100-199 µg/L. The two broken trendlines illustrate that s-Tg differed with TSH *only* when UIC was either above or below the recommended range for nonpregnant women, below 100 µg/L or above 199 µg/L.

The association between TSH and s-Tg differed between UIC groups. No significant association was seen between TSH and s-Tg in the group with UIC in the range of 100-199 µg/L (Figure 5, solid line), corresponding to WHO's definition of iodine sufficiency in pregnancy. In contrast, a statistically significant inverse association was seen in women with UIC <100 µg/L and in those with UIC ≥200 µg/L [dotted and dashed lines, respectively;  *P*= .03 (for interaction)].

## Discussion

In a cohort of Faroese pregnant women, s-Tg levels were raised only in the group with UIC below 50 µg/L, indicating that strain on the thyroid gland during pregnancy required UIC levels parallel to that of non-pregnant individuals.^24^ In addition, none of the participating pregnant women were identified with unknown thyroid dysfunction.

Our study results indicate that in this region, where the median UIC of pregnant women is below 150 μg/L, pregnant women are able to sustain iodine-related biological activity to uphold sufficient thyroid hormone secretion. Similarly, a study from China found that pregnant mothers with a median UIC of 107.4 µg/L, although below the WHO standard of 150 µg/L, were able to sustain sufficient iodine nutritional status for themselves and their newborns.^25^ While the median UIC is a common metric for evaluating a population's iodine status, the primary physiological role of iodine lies in its role in the creation of thyroid hormones.

The absence of untreated thyroid disease among the Faroese women referred for obstetric ultrasound and included in the study could be related to several factors. Our study population had free and easy access to general practitioners in support of early detection of subtle disease. Also, obstetric monitoring may have supported early detection of thyroid dysfunction during pregnancy and facilitated timely intervention among Faroese pregnant women. While the reported frequency of 15% with thyroid dysfunction in another population comprised of all types of abnormalities and hence included isolated changes in fT4 levels,^26^ such isolated changes in fT4 were not classified as thyroid disease in our population with normal TSH. This was chosen as the concept of isolated abnormal T4 is redundant.^27^ Finally, we lack knowledge of the occurrence of thyroid dysfunction among the one-in-three pregnant Faroese women who chose not to participate in the present study. Still, the absence of thyroid disease in a population of women with UIC classified as adequate for non-pregnant yet insufficient for pregnant women^7^ provided an opportunity to explore the strain on the thyroid gland imposed by pregnancy.

The use of iodine-containing supplements may have influenced the observed UIC levels. As reported previously,^28^ iodine-containing supplements increase UIC and may also contribute to greater inter-individual variability in spot urine samples. While first-morning voids are generally less affected by recent iodine intake compared with random spot samples, the timing and consistency of supplement use may still impact individual UIC measurements. The inclusion of supplement users in the cohort may therefore slightly elevate median UIC, obscure the relationship between habitual dietary iodine intake and UIC. This should be considered when interpreting the results.

Thyroglobulin concentration is a suitable marker of iodine nutrition status in a population.^12^ In pregnant women, s-Tg concentration was associated with iodine nutrition when the median UIC was below 100 μg/L,^29^ but not when UIC was above 120 μg/L.^30^ Specifically, in the groups with UIC <100 µg/L, the higher s-Tg levels were associated with lower TSH concentrations, suggesting a compensatory increase in thyroid activity with leakage of Tg into the circulation. A similar, though less pronounced, inverse association was observed in the group with UIC ≥200 µg/L. Thus, s-Tg in the present Faroese population may provide insight into the iodine intake group that has a burdened thyroid. We found higher s-Tg without thyroid dysfunction when UIC was below 50 µg/L. This rise in s-Tg was confirmed in the regression model using restricted cubic splines and conformed to an increased burden on the thyroid in this group. The finding of raised s-Tg suggesting strain on the thyroid gland is expected with ID. Interestingly, the similar s-Tg in the groups of pregnant women with UIC between 50-99 µg/L and 100-149 µg/L compared with UIC above 150 µg/L observed in our cohort suggests sufficient iodine intake with UIC below 150 µg/L. Thus, the range recommended by the WHO for pregnant women^7^ may be overly strict and re-evaluation of the lower limit of recommended UIC may be needed.

Iodine nutrition is evaluated by measuring UIC in groups of people^7^ as 90% of the ingested iodine is excreted in the urine.^8^ Numerous physiological changes occur in normal pregnancy, including haemodynamic changes and a marked rise in glomerular filtration rate (GFR),^31^ and increased thyroid hormone production to meet maternal and foetal needs. The pregnancy-induced rise in renal excretion also causes increased loss of nutrients in the urine.^32,33^ An estimated doubling of renal iodine clearance during pregnancy has been suggested^6^ but not confirmed.^34^ Although these changes occur, the need for a pregnancy-specific UIC threshold remains debated, as physiological variations in iodine excretion may not consistently reflect iodine status. Based on our results, we speculate that UIC does not require adjustments in pregnancy as GFR and urinary iodine excretion may increase proportionally. Such simultaneous increases preserve the UIC as a reliable biomarker for assessing iodine status during pregnancy, and we suggest that UIC of 100-199 μg/L designates an iodine-replete population also in pregnancy. Indeed, the simultaneous and parallel increase in iodine clearance and GFR would make the separate category for UIC in pregnancy redundant.

A strength of the present study is the nationwide data collection with a high response rate, which enhances the generalizability of the findings. Moreover, the combination of UIC, s-Tg, and TSH provided valuable insights into thyroid health and its relationship with iodine nutrition during pregnancy. A potential limitation of this study is the use of FMV urine samples for the assessment of UIC. While FMV sampling offers logistical advantages, particularly in the context of routine antenatal care, previous studies have shown that UIC in FMV samples may be 10%-20% lower than in spot samples collected later in the day, likely due to the overnight fasting state.^35-39^ This should be considered when interpreting UIC results. Non-pregnant reference intervals were reported for free T4, as pregnancy-specific reference ranges are not established for this population. However, reference ranges were not used for the interpretation of results, as T4 was included only in the analysis of associations. Still, recall and selection bias may be present, but the influence on the results is uncertain and expected to be random with limited influence on the results. The sample size calculation recommended a minimum of 206 serum samples to achieve a precision of 10% for s-Tg with a 95% confidence. This sample size was set to estimate the median serum Tg concentration with adequate precision, while the association between UIC and s-Tg was an additional analysis, which was beyond the sample size calculation. This regression model using splines could underestimate the true mean, but the low model variance indicates that this underestimation is negligible. Thyroglobulin antibody could cause interference in the laboratory analysis but was not measured. These could influence Tg levels by raising the variance but should have limited influence on mean s-TG. The assessment of iodine nutrition from a single spot urine sample is a limitation, as a single sample does not reflect long-term iodine status.^40^ A longitudinal follow-up on thyroid function and iodine nutrition throughout pregnancy would improve the understanding of the relationship between iodine intake and thyroid health, and ultrasonic examinations of the thyroid could add valuable insights.

## Conclusion

This study offers novel insights into the iodine and thyroid health of pregnant women. While supporting the importance of monitoring and promoting adequate iodine nutrition during pregnancy, we speculate whether the current cut-off for UIC recommended by WHO should be reconsidered and possibly lowered. Our findings contribute to the growing body of evidence suggesting that current WHO recommendations for UIC thresholds during pregnancy may warrant re-evaluation. The association with rising s-Tg levels only in the group with UIC below 50 μg/L supports aligning recommendations for pregnant women with those for non-pregnant adults and balancing the efficacy and simplicity of guidelines. Finally, exploring the relationship between maternal s-Tg levels and neonatal TSH levels could add another approach to provide a clearer understanding of the optimal iodine intake during pregnancy.

## Data Availability

Study data cannot be shared due to regulatory restrictions that apply to the availability of data generated and analysed during this study to preserve patient confidentiality and according to the GDPR.
